# Caregiver strain modulates the association between attention deficit and alpha oscillations in children with ADHD

**DOI:** 10.3389/fpsyt.2026.1811824

**Published:** 2026-04-22

**Authors:** Xiangsheng Luo, Mengqi Liu, Hanbo Zhang, Junhui Qi, Yanjie Qi, Huanhuan Huang, Yiwei Lin, Yin Tian, Lichong Luo, Tianyu Qu, Li Qin, Longjun Cai, Li Sun, Xiaojie Guo, Xu Chen

**Affiliations:** 1 Beijing Key Laboratory of Intelligent Drug Research and Development for Mental Disorders; National Clinical Research Center for Mental Disorders; National Center for Mental Disorders; Beijing Anding Hospital, Capital Medical University, Beijing, China; 2 Department of Medical Psychology, the First Medical Center, Chinese People's Liberation Army (PLA) General Hospital, Beijing, China; 3 Beijing Wispirit Technology Co., Ltd, Beijing, China; 4 Peking University Sixth Hospital & Peking University Institute of Mental Health, Child Psychiatry, Beijing, China

**Keywords:** ADHD, alpha oscillations, caregiver strain, developmental psychopathology, mediation effect

## Abstract

**Introduction:**

The neurobiological mechanisms underlying Attention-Deficit/Hyperactivity Disorder (ADHD) remain incompletely understood. Existing research has identified abnormalities in alpha rhythm among individuals with ADHD; however, its association with core symptoms lacks consistency, suggesting that enhanced alpha activity may represent a state-dependent compensatory manifestation. The family environment, particularly caregiver stress, is recognized as an important external factor influencing the development of children with ADHD, yet its potential role as a mediator between clinical symptoms and neural brain activity has yet to be systematically explored.

**Methods:**

The study included 59 children with ADHD. Correlations among attention deficit scores (ADS), hyperactivity/impulsivity scores (HIS), various dimensions of caregiver strain, and posterior alpha power were analyzed, with Bonferroni correction applied to control for multiple comparisons. Subsequently, hierarchical regression and mediation modeling were employed to examine the mediating effect of caregiver strain.

**Results:**

No direct correlation was found between ADS and alpha power. A triangular pattern among symptoms, stress, and brain activity emerged: ADS showed strong positive correlations with all dimensions of caregiver strain, and subjective internalized strain (SIS) remained significantly positively correlated with alpha power even after correction. The mediation model indicated a suppression pattern, wherein the statistical association between ADS and alpha power was consistent with a positive indirect pathway via SIS, alongside a masked direct association, resulting in a non-significant total effect.

**Discussion:**

The findings suggest a model in which SIS is a key statistical mediator in the relationship between ADHD symptoms and specific neural oscillatory patterns, and highlight the plasticity of brain function in response to the family emotional environment.

## 1 Introduction

Attention-Deficit/Hyperactivity Disorder (ADHD) is one of the most common neurodevelopmental disorders, affecting approximately 5% of children and adolescents worldwide (1). It is characterized by a persistent pattern of inattention, hyperactivity, and impulsivity, which significantly impairs academic achievement, social functioning, and occupational performance. The disorder often persists into adulthood, leading to substantial lifelong personal distress and considerable socioeconomic burden, with annual costs estimated at hundreds of billions of dollars (2). Despite its high prevalence and significant impact, the precise etiology and underlying neurobiological mechanisms of ADHD remain incompletely understood, and no reliable objective biomarker has been established for clinical diagnosis.

Electroencephalography (EEG) has been widely used to investigate the neural underpinnings of Attention-Deficit/Hyperactivity Disorder (ADHD). Among various EEG metrics, spectral power and functional connectivity within the alpha frequency band (8–13 Hz) have garnered considerable attention. The alpha rhythm, the dominant oscillatory activity in the awake, resting human brain, reflects the brain’s arousal state and plays a crucial role in attentional control (3–5). Current research suggests that the alpha rhythm can be utilized to explore the neurophysiological characteristics of ADHD (6).

A substantial body of research has documented atypical alpha-band activity in individuals with ADHD, while the findings exhibit significant heterogeneity. Some studies report reduced alpha power in ADHD, interpreted as a marker of cortical hypoarousal, yet found no association between alpha power and core ADHD symptoms (7). Conversely, other studies have found elevated alpha power in children with ADHD compared to typically developing controls (8). Our previous research adds to this complex picture: a longitudinal study showed that cognitive training led to improved attentional symptoms alongside a significant reduction in resting-state alpha power in children with ADHD, although a direct correlation between symptom change and alpha power change was not established (9). In another study, we found that enhanced alpha power in ADHD may function as a compensatory mechanism rather than a primary feature of the disorder (10). And, studies have identified an ADHD subtype characterized by alpha hyperactivation associated with emotional problems, aberrant alpha activity in ADHD may be linked to dysregulation of the autonomic nervous system’s stress response (11). This recurring “neural-behavioral dissociation” suggests that elevated alpha activity in ADHD may not be a direct pathological product of inattention but is more likely a state-dependent manifestation of neural regulation.

Crucially, the neural dysregulation itself is highly susceptible to influence from external environmental factors. For children with ADHD, the family environment serves as a vital external factor modulating and influencing their patterns of brain functional development (12). First, the behavioral manifestations of children with ADHD significantly increase parental stress, which in turn affects the family atmosphere and parenting styles (13). Strained family relationships also elevate stress levels in primary caregivers (14), while children’s problematic behaviors further exacerbate caregivers’ stress and family instability (15). Parents under stress are more likely to exhibit negative or controlling parenting behaviors, fostering a high-pressure emotional climate within the family (16).

This family dysfunction can lead children to experience heightened anxiety and stress, manifested as excessive cortisol secretion, thereby influencing brain development (17). Such chronic stress may affect functional developmental stages, including the hypothalamic-pituitary-adrenal (HPA) axis and the noradrenergic system, inducing structural and functional alterations in key brain regions responsible for learning, cognition, emotional regulation, and executive function (18). Consequently, this exerts sustained negative effects on child development (19), such as prominent emotional and behavioral symptoms (20) and unfavorable prognostic outcomes (21–23), ultimately having a lasting impact on children’s brain functional development. Therefore, caregivers’ stress levels may act as a pivotal mediating factor linking ADHD symptoms to brain functional development in children.

Based on the integrated perspective outlined above, we propose a novel mediating model: family environmental factors, particularly caregiver stress, serve as a critical mediating link connecting inattentive symptoms in children with ADHD to aberrant alpha EEG activity. The proposed pathway is as follows: more severe inattentive symptoms → higher levels of subjective caregiver stress → inducing or sustaining a compensatory enhancement of posterior alpha activity in the child. This study provides, for the first time from a family systems perspective, mechanistic evidence for understanding the heterogeneity of neurophysiological markers in ADHD and their interaction with environmental factors.

## 2 Method

### 2.1 Participants

This study adopted a cross-sectional design. Participants were children with ADHD recruited from Beijing Anding Hospital, Capital Medical University. A total of 59 children with ADHD were enrolled, with a mean age of 9.124 ± 1.929 years, including 43 boys and 16 girls, all participants were not undergoing systematic medication treatment for ADHD at the time of enrollment. The study protocol was approved by the Ethics Review Committee of Beijing Anding Hospital, Capital Medical University (ID: 2023 Keyan No. 328-202418FS-2), and written informed consent was obtained from the legal guardians of all children prior to their participation. Research data were collected using the Electronic Data Capture (EDC) system.

### 2.2 Inclusion and exclusion criteria

Children in the ADHD group were diagnosed by experienced psychiatrists according to the criteria of the Diagnostic and Statistical Manual of Mental Disorders, Fifth Edition(DSM-5). The inclusion criteria were as follows: (1) diagnosis of ADHD by a psychiatrist at the attending physician level or above, confirmed as meeting DSM-5 diagnostic criteria; (2) age between 6 and 16 years, irrespective of sex, with parents willing to participate in the study as required; and (3) a Raven’s Progressive Matrices score ≥ 25%. The exclusion criteria included: (1) significant physical or neurological abnormalities, or notable hearing or visual impairment; and (2) comorbid psychiatric disorders such as childhood-onset schizophrenia, mood or affective disorders, intellectual disability, or autism spectrum disorder.

### 2.3 Materials

#### 2.3.1 Clinical symptom assessment scale

Attention-Deficit/Hyperactivity Disorder Rating Scale (ADHD-RS):​The ADHD-RS was developed based on the diagnostic criteria for ADHD in the DSM. It consists of 18 items rated by the child’s primary caregiver according to the frequency of symptomatic behaviors, using a 4-point scale from 0 to 3. The first 9 items assess inattention symptoms, with their total score forming the Inattention Score (ADS); the remaining 9 items assess hyperactivity-impulsivity symptoms, with their total score forming the Hyperactivity-Impulsivity Score (HIS). This scale serves as a primary instrument for assessing the core symptoms of ADHD.

#### 2.3.2 Parenting stress assessment

Caregiver Strain Questionnaire (CGSQ):​ Developed by Brannan and colleagues from McLean Hospital in 1997, the CGSQ is designed to assess parenting stress among parents of children under 18 years old (24). It is a commonly used, reliable, and valid measure of caregiver strain (25). This scale employs subjective ratings provided by the parents of the patients. Based on the focus of the strain reflected in the assessment, it is categorized into three dimensions, which are named according to the substantive content reflected by each dimension: (1) Objective Strain (OS), which refers to observable negative events resulting from the child’s problems (e.g., financial strain, disruption of family relationships; 11 items); (2) Subjective Internalized Strain (SIS), which measures inwardly directed negative feelings experienced by the caregiver due to the child’s problems (e.g., sadness, worry; 6 items); and (3) Subjective Externalized Strain (SES), which assesses negative feelings directed toward the child (e.g., anger, resentment; 4 items). The scale was demonstrated high reliability and validity (26).

#### 2.3.3 Neurophysiological data acquisition

Resting-state electroencephalography (EEG) data were acquired using a 128-channel HydroCel Geodesic Sensor Net (Electrical Geodesics, Inc.) and Net Station software. Approximately six minutes of EEG were recorded under eyes-open conditions. The online reference was set to Cz, and electrode impedance was maintained below 50 kΩ. The signals were amplified with a band-pass filter of 0.01–400 Hz and digitized online at a sampling rate of 1000 Hz.

**Figure 1:**
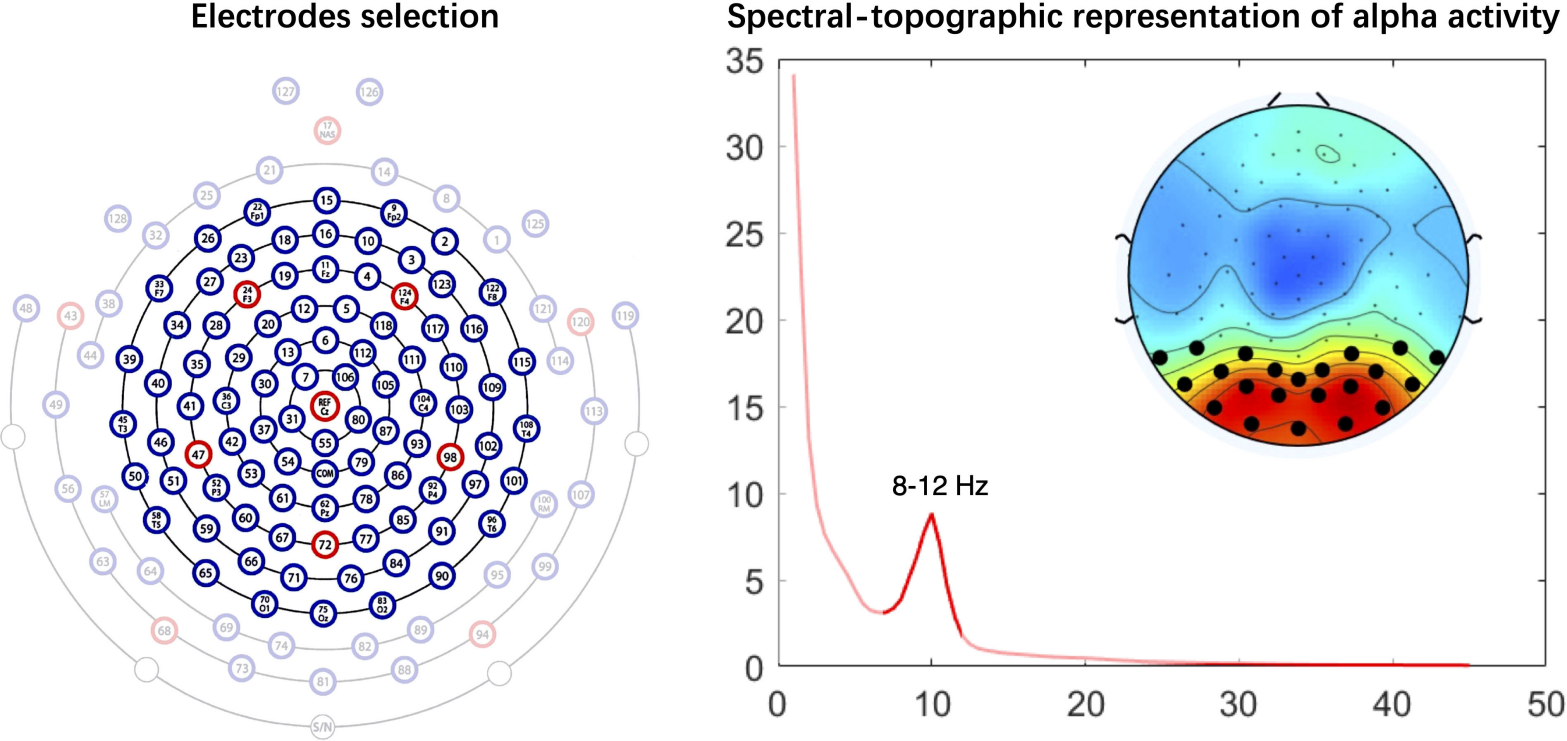
EEG analysis results. Left panel: Schematic diagram of the 128-channel electrode array, data from 91 central electrodes were primarily extracted. Right panel: The topographic map displays the spatial distribution of absolute alpha power (8–12 Hz) across the scalp. The power spectral density plot derived from the selected posterior electrodes (black dots) during the eyes-open resting state.

The EEG analysis method followed our previous research protocol. For children with ADHD, data from 91 central electrodes were primarily extracted, excluding 38 peripheral electrodes that are more susceptible to movement and muscular artifacts (see **Figure 1**). The data were resampled to 250 Hz and band-pass filtered between 1 and 45 Hz. Artifacts were manually rejected, and bad channels were interpolated. Independent Component Analysis (ICA) was performed to identify and remove components associated with vertical and horizontal eye movements. Spectral analysis was conducted using the pwelchfunction on 2-second epochs. Absolute alpha power (8–12 Hz) was extracted from posterior electrodes (20 electrodes) based on the alpha topographic map (see **Figure 1**). For each child, the average absolute power from the selected electrodes was calculated and used for subsequent statistical analysis.

### 2.4 Statistical analysis

All data analyses were performed using SPSS 22.0 and the PROCESS v3.0 macro. The analysis consisted of three parts.

#### 2.4.1 Preliminary analysis

Descriptive statistics and normality tests were conducted. Pearson correlations were used to examine the bivariate associations between core clinical symptoms (ADS, HIS), caregiver strain (OS, SIS, SES), and alpha power, with age and sex was controlled as covariates. To control for the inflation of Type I error due to multiple comparisons, a Bonferroni correction was applied to all correlation analyses. The corrected significance level (α_corrected) was calculated based on the number of tests (k) included in each analysis (α_corrected = 0.05/k).

#### 2.4.2 Hierarchical regression analysis

A hierarchical regression analysis was conducted to examine the influence of caregiver stress on alpha power, with alpha power serving as the dependent variable. Predictors were entered in three blocks: Block 1 (Model 1) included the demographic variables sex and age; Block 2 (Model 2) added the core ADHD symptom scores (ADS and HIS); and Block 3 (Model 3) introduced the caregiver stress variable (SIS), which had shown a statistically significant correlation with alpha power.

For the regression analysis, the categorical variable “sex” was coded as a dummy variable, with female as the reference category. Sex was included given the heterogeneous distribution of boys and girls in the sample and the well-documented sex differences in ADHD presentation. Age was also included as a covariate because alpha power demonstrates significant developmental changes across childhood and adolescence, and ADHD symptomatology evolves with age (e.g., inattention often persists while hyperactivity-impulsivity may decline).

#### 2.4.3 Mediation analysis

A mediation analysis was performed using a Bootstrap-based approach (PROCESS Model 4) to test the potential mediating role of caregiver stress in the relationship between ADHD symptoms and alpha activity. Sex and age were included in the model to control for their potential confounding effects. The indirect and total effects were tested by generating 5000 Bootstrap samples and calculating the 95% bias-corrected confidence intervals (CI). A significant indirect effect (mediation) was inferred if the CI did not contain zero. The significance level was set at α = 0.05.

## 3 Results

### 3.1 Preliminary analysis

Descriptive analyses were conducted on a final sample of 59 participants and showed in the **Table 1**. The absolute values of skewness (range: 0.18 to 1.25) and kurtosis (range: -0.84 to 1.79) for all variables were below 2, indicating that the data did not severely deviate from the normality assumption, thus satisfying the prerequisites for subsequent parametric analyses (27).

**Table 1:** Descriptive statistics.

Variables	Mean ± SD	Q1, Q3	Skewness	Kurtosis​
Age	9.124 ± 1.929	7.667, 10.500	0.974	1.156
Sex (M/F)	43/16		–	–
ADS	16.542 ± 4.340	14, 19	0.180	-0.690
HIS	10.661 ± 6.048	6, 16	0.360	-0.550
OS	22.186 ± 8.713	17, 27	0.240	-0.840
SIS	13.475 ± 5.302	9, 16	0.320	-0.770
SES	8.068 ± 3.162	6, 10	0.190	-0.630
Alpha	21.980 ± 18.306	7.670, 29.834	1.254	1.791

ADS, Attention deficit scores; HIS, Hyperactivity/Impulsivity scores; OS, Objective strain; SIS, Subjective internalized strain; SES, Subjective externalized strain; SD, Standard Deviation; M, Male; F, Female; Interquartile range (IQR; Q1, Q3); Alpha, posterior resting-state alpha power (in dB).

**Figure 2:**
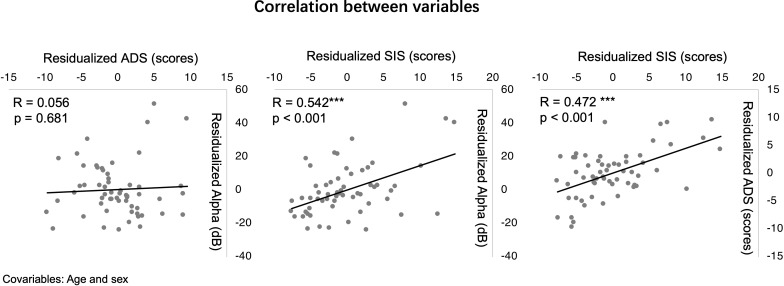
Scatterplots illustrating the bivariate correlations among residualized scores of attention deficit scores (ADS/scores), caregiver subjective internalized strain (SIS/scores), and posterior alpha power (Alpha/dB), with age and sex as covariables. ***p < 0.001.

The first step involved generating a correlation matrix, with Pearson correlation coefficients and p-value presented in **Table 2**. To control for the inflation of Type I error due to multiple comparisons, a Bonferroni correction was applied, setting a stringent significance threshold at α = 0.003 (adjusted for 15 comparisons). Notably, ADS showed strong positive correlations with all three dimensions of caregiver strain: OS (r = 0.483, p < 0.001), SIS (r = 0.609, p < 0.001), and SES (r = 0.542, p < 0.001), indicating that more severe inattentive symptoms in children were associated with greater objective and subjective caregiver stress. Pearson correlation analyses revealed one key cross-construct association that survived this strict correction for the Alpha power: SIS was significantly and positively correlated with Alpha power (R = 0.472, p <0.001). This indicates that higher levels of caregivers’ inwardly directed negative emotions (e.g., worry, sadness) were associated with greater alpha oscillatory activity in the posterior brain regions of children with ADHD. This pattern provided preliminary support for the hypothesized mediating role of caregiver strain in the link between ADHD attention deficits and specific alpha activity (see **Figure 2**).

**Table 2:** Pearson correlations.

	ADS	HIS	OS	SIS	SES	Alpha
ADS	*R*	0.337**	0.483***	0.542***	0.609***	0.056
	*p*	0.010	**< 0.001**	**< 0.001**	**< 0.001**	0.681
HIS		*R*	0.208	0.194	0.281*	0.109
		*p*	0.120	0.148	0.034	0.418
OS			*R*	0.824***	0.730***	0.353
			*p*	< 0.001	< 0.001	0.007
SIS				*R*	0.839***	0.472***
				*p*	< 0.001	**< 0.001**
SES					*R*	0.360**
				*p*	0.006

ADS, Attention deficit scores; HIS, Hyperactivity/Impulsivity scores; OS, Objective strain; SIS, Subjective internalized strain; SES, Subjective externalized strain; M, Mean; SD, Standard Deviation. Values in bold indicate correlations that remained significant after Bonferroni correction (adjusted α = 0.00333). Correlations among the three caregiver strain subscales (OS, SIS, SES) are presented for completeness but are not emphasized as they measure facets of the same construct.

* *p* < 0.05; ** *p* < 0.01, *** *p* < 0.001.

Several other correlations were significant at the conventional p < 0.05 level but did not survive the Bonferroni correction. HIS was positively correlated only with SES (r = 0.281, p = 0.034). Furthermore, OS (r = 0.353, p = 0.007) and SES (r = 0.360, p = 0.006) also showed positive correlations with Alpha power, although these did not reach the corrected significance threshold. Crucially, no significant direct correlation was found between the core symptom of attention deficit (ADS) and Alpha power (r = 0.056, p = 0.681).

As expected, the three subscales of the caregiver strain questionnaire (OS, SIS, and SES), which measure facets of the same underlying construct, were highly intercorrelated (rs ranging from 0.730 to 0.839, all ps < 0.001). These correlations are reported for completeness but are not emphasized in the interpretation.

### 3.2 Hierarchical regression analysis

In the second step of the analysis, we employed hierarchical multiple regression to examine the predictive effects of demographic variables, ADHD symptoms, and caregiver strain on resting-state alpha EEG power. Specifically, Block 1 (Model 1) included age and sex. Block 2 (Model 2) added the scores of the ADS and HIS subscales from the ADHD-Rating Scale to the previous model. Block 3 (Model 3) further introduced caregiver SIS, which showed a significant correlation with alpha power.

The results indicated that all three regression models were statistically significant. Model 1 significantly predicted alpha power, *F*(2, 56) = 8.799, *p* < 0.001. Model 2 also showed significant predictive power, *F*(4, 54) = 4.465, *p* = 0.003. The predictive power was further enhanced in Model 3, *F*(5, 53) = 8.943, *p* < 0.001.

As shown in **Table 3**, in Model 1, which included only demographic variables, sex was a significant predictor of alpha power (*B* = -20.049, *SE* = 4.808, β = -0.491, *p* < 0.001), indicating that male children had significantly higher alpha power. This model explained 23.9% of the variance in the dependent variable. In Model 2, which incorporated core ADHD symptoms, sex remained a significant predictor, while neither the ADS nor HIS symptoms reached significance. The overall explanatory power of Model 2 (*R*² = 0.249) showed only a slight and non-significant increase compared to Model 1 (Δ*R*² = 0.009).

**Table 3:** Regression models for the prediction of resting-state alpha power.

	Model 1		Model 2		Model 3	
	B (β)	*t*	B (β)	*t*	B (β)	*t*
Age	-0.157(-0.017)	-0.140	0.021 (0.002)	0.018	-0.545 (-0.057)	-0.531
Sex	-20.049 (-0.491)	-4.170***	-19.754 (-0.484)	-4.016***	-18.035 (-0.442)	-4.258***
ADS			0.079 (0.019)	0.147	-1.168 (-0.277)	-2.186*
HIS			0.278 (0.092)	0.712	0.335 (0.085)	0.767
SIS					1.911 (0.553)	4.520***
*R* ^2^	0.239		0.249		0.458	
Δ*R*^2^			0.009		0.209***	

The values in the table are non-standardized β coefficients; standardized values are given in parentheses. *t* = Student t-test; *R*^2^ = variance explained; Δ*R*^2^ = change in variance explained.

* *p* < 0.05; *** *p* < 0.001.

Finally, in Model 3, which included SIS emerged as a significant positive predictor of alpha power (B = 1.911, *SE* = 0.423, β = 0.553, *p* < 0.001), and the standardized beta coefficients indicated that, within this sample, caregiver SIS was the strongest predictor of children’s resting-state alpha power. In addition, when controlling for SIS, the predictive effect of ADS on alpha power turned into a significant negative predictor (B = -1.168, SE = 0.534, β = -0.277, p = 0.033), whereas its effect in Model 2 was not significant (B = 0.019, p = 0.883). This change suggests that SIS may modulate and influence the interaction between alpha power and ADS. The effect of sex, though still significant, was reduced in magnitude (*B* = -18.035, *SE* = 4.235, β = -0.442, *p* < 0.001). This final model explained 45.8% of the variance in resting-state alpha power. The increase in explanatory power compared to Model 2 (Δ*R*² = 0.209) was highly statistically significant (*p* < 0.001).

### 3.3 Mediation effect analysis

The rigorous correlation and hierarchical regression analyses described above revealed a specific association pattern among alpha activity, clinical symptoms, and the family environment in children with ADHD: (1) The alpha activity of children with ADHD was significantly influenced by SIS; (2) SIS was closely related to the ADS of ADHD; (3) SIS may modulate the interaction between alpha power and ADS; (4) Sex played a significant role in the alpha activity of children with ADHD. This pattern suggests that SIS may serve as a critical link between clinical symptoms and specific neural activity patterns. To further investigate whether this link represents a moderating or mediating effect and to clarify the causal pathways among the variables, we conducted a mediation effect analysis.

To test whether SIS mediates the relationship between ADS and the absolute alpha power at posterior electrodes in children with ADHD, we employed the bias-corrected percentile Bootstrap method (with 5, 000 resamples) to test the significance of the indirect effect, with sex was controlled as a covariate.

The results revealed a clear pattern of suppression. First, the path tests showed that ADS had a significant positive predictive effect on the mediator SIS (Path a: B = 0.658, SE = 0.137, t = 4.785, p < 0.001). Simultaneously, SIS had a significant positive predictive effect on the outcome variable Alpha (Path b: B = 1.915, SE = 0.421, t = 4.548, p < 0.001). The total effect of ADS on Alpha was not significant (Total effect c: B = 0.207, SE = 0.500, t = 0.414, p = 0.681, 95% CI [-0.795, 1.209]), while its direct effect (Path c’) showed a significant negative effect when SIS was controlled (B = --1.053, SE = 0.511, t = -2.061, p = 0.044, 95% CI [-2.076, -0.029]).

**Figure 3:**
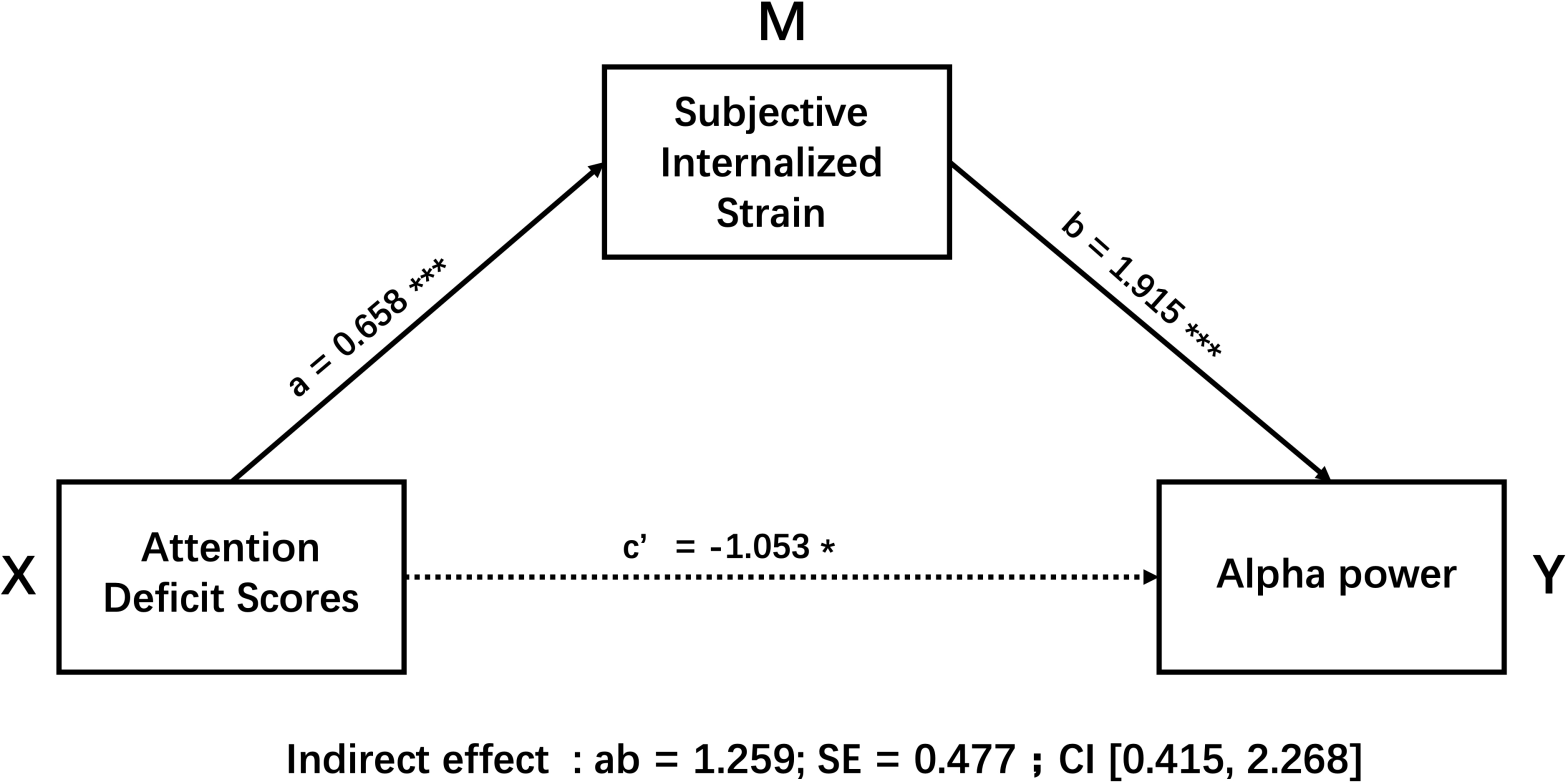
The mediation model showing the suppressive role of SIS(M) in the relationship between ADS(X) and Alpha(Y), controlling for age and sex. Unstandardized regression coefficients are presented. Paths a and b are significantly positive (p < 0.001), while the direct path c’ is significantly negative (p < 0.05). The indirect effect (ab) is significant, with a point estimate of 1.259 and a 95% bootstrap confidence interval of [0.415, 2.268] not including zero. The total effect c was non-significant (p > 0.05). * p < 0.05; *** p < 0.001.

The Bootstrap test for the indirect effect provided key evidence for interpreting this apparent contradiction. The indirect effect of ADS on Alpha through SIS was 1.259, with a 95% Bootstrap confidence interval of [0.415, 2.268], which does not include zero, indicating that this indirect effect is statistically significant. This result confirms the coexistence of a significant positive indirect path and a significant negative direct path, jointly resulting in a non-significant total effect—a statistical characteristic of a suppression effect (see **Figure 3**).

In summary, after controlling for sex, this study found robust evidence for a suppression effect of SIS on the relationship between ADS and Alpha. ADS exerts a positive indirect effect on Alpha by increasing the level of SIS, while it shows a negative direct effect trend on the outcome variable. These opposing effects cancel each other out, ultimately causing the total effect to remain non-significant.

## 4 Discussion

This study integrated data on clinical symptoms - ADS, family stress - SIS, and brain function – posterior Alpha power with eyes open, systematically revealing a statistical model that underscores​ the potential role of the family system in the “behavior-brain” association in ADHD. Findings showed no direct link between ADS and Alpha, but both were robustly and positively correlated with SIS, forming a symptom-stress-brain activity triangular pattern. Mediation analysis further identified a significant suppression effect of SIS, indicating a statistical pattern where​ symptoms showed a positive indirect association with Alpha activity by increasing caregiver stress, while their direct effect was masked. This pattern is consistent with the view that enhanced posterior Alpha activity in children with ADHD may not be a fixed endophenotype but rather a state-dependent correlate, potentially modulated by​ the psychosocial family environment, particularly caregiver stress.

### 4.1 Association between ADHD symptoms and elevated caregiver stress

The behavioral symptoms of ADHD, particularly attention deficit, are associated with elevated stress levels among caregivers, intensifying feelings of anxiety, tension, and frustration (i.e., subjective internalized strain) experienced during parenting. This finding is consistent with prior research indicating that ADHD adversely affects the family system (28). ADHD is associated with a range of unmet needs for both individuals and their families (29), and strained family dynamics significantly exacerbate negative emotions in caregivers Chang and Chiang 2022). Specifically, ADHD symptoms are a significant predictor of depressive symptoms in caregivers (30), who also report heightened anxiety concerning their child’s well-being (31). The severity of stress experienced by parents of children with ADHD is well-documented, underscoring the necessity for targeted interventions (13).

Regarding the two core symptom domains of ADHD, this study found that ADS)showed strong and consistent positive correlations with all dimensions of caregiver strain, whereas the association between HIS and strain was weaker and less robust. Specifically, HIS was significantly correlated only with Subjective Externalized Strain (SES, reflecting caregivers’ negative evaluations toward the child, e.g., feeling angry or resentful) (r = 0.281, *p* = 0.034), an association that did not survive stringent multiple comparison correction. This suggests that while HIS symptoms may also contribute to elevated caregiver strain, their association is substantially weaker compared to that of ADS.

This pattern partially diverges from the clinical intuition that hyperactive/impulsive behaviors, due to their more overt and disruptive nature, might be a stronger driver of caregiver stress. Potential explanations for this discrepancy are twofold. First, interviews indicate that caregivers are often particularly sensitive to academic stress, which is a frequent primary complaint leading to clinical consultation (32). Research suggests that ADS independently predicts academic performance in children with ADHD, whereas the predictive power of HIS is less stable (33). Therefore, within cultural contexts that emphasize academic achievement, ADS symptoms may more readily attract caregiver concern and subsequently elevate strain. Second, this finding relates to the clinical characteristics of our sample. As shown in **Table 1**, the mean HIS score (10.661 ± 6.048) was lower than the mean ADS score (16.542 ± 4.340), indicating that our sample was predominantly characterized by significant inattention. Thus, the behavioral challenges and associated stress risks specifically linked to HIS might be less pronounced in this group.

### 4.2 Association between alpha activity and caregiver strain

On the other hand, the study shows that SIS is significantly positively correlated with alpha activity activation in children with ADHD. Furthermore, our regression analysis indicates that SIS is a significant influencing factor for alpha activity in children with ADHD. Specifically, caregivers’ negative emotions (such as anxiety, tension, and dissatisfaction) are associated with elevate alpha activity levels in children with ADHD.

Research has established a stable association between anxiety and alpha-band EEG activity. Further mechanistic studies on state and trait anxiety reveal an enhancement in alpha synchronization: anxiety can increase an individual’s level of alpha synchronization, thereby heightening alertness and enhancing autonomic nervous system preparedness. Consequently, individuals with high anxiety exhibit greater alpha power and also show enhanced alpha desynchronization during tasks (34, 35). Simultaneously, anxious traits have been confirmed to predict changes in alpha power between attention and resting states, with higher anxiety levels correlating with greater alpha disparity. This suggests a close link between changes in alpha activity and anxiety states (36). Among children with ADHD, elevated alpha activity has also been found to potentially reflect a subgroup of children with emotional dysregulation (11).

Theoretically, an increase in caregivers’ negative emotional stress could be related to the adoption of harsh yet ineffective parenting styles and difficulties in setting boundaries, all of which may deteriorate parent-child relationships and contribute to the development of insecure attachment (37). A high-pressure family environment exerts a sustained negative impact on children’s emotional and behavioral development (19, 20), potentially leading to an overall increase in family stress, stricter disciplinary approaches, or a more unstable family rearing atmosphere. This may further trigger a series of abnormal physiological and psychological changes (38), ultimately increasing the risk of internalizing emotional dysregulation (39) and somatic autonomic dysfunction in children (40). In such an environment, children are also more likely to experience excessive scrutiny or control, thereby exhibiting more pronounced autonomic dysregulation. The process of highly aroused autonomic activation manifests as a significant elevation in alpha activity.

### 4.3 The statistical mediation model for caregiver strain, symptoms and alpha activity

In summary, while no direct association was found between alpha activity and inattention symptoms in children with ADHD, both were significantly correlated with caregivers’ subjective strain, suggesting a potential mediating model. Mediation analysis confirmed this mechanism: more severe inattention symptoms led to higher subjective strain, which in turn exerted a positive indirect promoting effect on the alpha power of children with ADHD. Concurrently, inattention symptoms themselves showed an independent, negative direct effect trend on EEG activity. These opposing positive and negative effects canceled each other out, resulting in the statistical illusion of no association in simple correlation analyses—a pattern indicative of a significant mediating pathway, characteristic of a suppression effect.

Although the present study did not observe a direct link between alpha activity and inattention symptoms, their relationship has been extensively debated in prior research, often with inconsistent findings (7). Some studies further subdivided alpha activity by frequency band, revealing associations with different ADHD symptom profiles or identifying ADHD subtypes with distinct alpha activity patterns (11). However, the replicability and stability of such results remain questionable. As an objective physiological measure, if alpha activity reflected a core deficit in ADHD, a stable association would be expected—a contradiction to the current lack of consistent correspondence in the literature. These opposing statistical effects resulted in the pattern of​ no association in simple correlation analyses—a classic suppression effect.

This study reveals a statistical mediation pattern centered on caregiver strain, which is consistent with a pathway where attention deficit symptoms are indirectly associated with enhance alpha activity in posterior brain regions by increasing caregivers’ subjective stress. This finding holds significant theoretical value as it supports the potential role of the family psychosocial environment between symptoms and physiological expression. It also suggests the view that the specific EEG patterns may be not static labels for neurodevelopmental disorders, as the​ enhanced posterior alpha activity may represent a state-dependent neural correlate of a negative family emotional climate. We propose that this model encourages a shift from a pure biological deficit model toward an integrated biopsychosocial framework for understanding ADHD.

Furthermore, this discovery suggests that future research on neurodevelopmental disorders should proactively incorporate assessments of family environmental factors—such as collecting parental mental health indicators or focusing longitudinal studies on younger children less exposed to prolonged family environmental influences—to more accurately isolate the disorder’s inherent neural characteristics. Clinically, our model implies that​ family-centered interventions, like parent stress management, might promote improvement in part by modulating the family emotional environment, a hypothesis worthy of future testing in intervention studies with EEG measures.

### 4.4 Limitations

This study has several limitations. First, the cross-sectional design precludes definitive causal inferences. Second, the lack of subtype differentiation and a broad range of survey participants was insufficient to support exploring more complex moderated mediation models. Third, the current findings remain primarily based on subjective assessment methods. The study lacks supporting objective physiological and monitoring data, for example, measures of the child’s arousal state during EEG recordings, broader psychological states of the caregivers, and objective evaluations of family interactions. Additionally, the assessment of other relevant child conditions such as sleep, emotional state, and family socioeconomic circumstances was insufficient. Future research could employ longitudinal designs and incorporate more objective measures of stress and more nuanced brain network analyses. This will be essential to move from the compelling statistical model presented here to a confirmed understanding of the dynamic interplay between child symptoms, family emotional processes, and brain function in ADHD.

## 5 Conclusion

In summary, the statistical mediation model observed in this study is consistent with the hypothesis​ that the family system, particularly caregiver subjective internal strain, plays a key role in the association between inattention symptoms and posterior alpha EEG activity in children with ADHD. The identified “suppression effect” provides a novel statistical framework for understanding the complex and often inconsistent relationship between clinical symptoms and neurophysiological measures in ADHD. This finding supports the view​ of significant environmental plasticity in ADHD-related brain function, highlighting that neural patterns may reflect state-dependent adaptations to the environmental context. Consequently, understanding and intervening in ADHD necessitates placing the child within the core context of their family relationships, shifting from a purely “brain disorder” perspective toward a dynamic “individual-environment” interactive systems view.

## Data Availability

The raw data supporting the conclusions of this article will be made available by the authors, without undue reservation.
